# Editorial: Redox Potential and Metabolic Behavior in Gastrointestinal Cancers

**DOI:** 10.3389/fonc.2022.882237

**Published:** 2022-04-27

**Authors:** Alessio Biagioni, Sara Peri, Nicola Schiavone, Elisa Giommoni, Laura Papucci

**Affiliations:** ^1^ Department of Experimental and Clinical Biomedical Sciences “Mario Serio”, University of Florence, Firenze, Italy; ^2^ Department of Experimental and Clinical Medicine, University of Florence, Firenze, Italy; ^3^ Medical Oncology Unit, Department of Oncology and Robotic Surgery, AOU Careggi, Firenze, Italy

**Keywords:** gastric cancer, colorectal cancer, gastrointestinal cancers, metabolism, redox potential, chronic hepatitis, tumor microenvironment

Gastrointestinal cancers refer to a large and heterogeneous family of malignancies localized in the esophagus, stomach, liver, pancreas, small and large intestine, rectum and anus that account for over 5,000,000 new cases per year and about 3,540,000 deaths worldwide (1). Characterized by very different diffusion between Eastern and Western countries, several factors influence the epidemiological distribution including environmental risk factors, prevention strategies and lifestyles. However, these histotypes of cancers are now widely recognized as very heterogeneous tumors (2), increasing the difficulty to find appropriate and efficient treatment regimens (3). One of the recently updated Hanahan’s hallmarks of cancer (4) is represented by dysregulation in cellular energetics and metabolism. This aspect was initially hinted at by the well-known Warburg effect for which hyper-proliferating cells showed accelerated conversion of glucose to lactate even in the presence of abundant oxygen in order to sustain energetics demand (5). This generates an abundant lactate secretion leading to rapid extracellular acidification which might often pair with an increased tumor migration, invasion, vascularization, chemoresistance and in the last metastasization (6) whilst the typical mitochondrial oxidative metabolism is inhibited also due to severely damaged mitochondria (7). This concept evolved in time not only for glucose metabolism, indeed alterations in several biochemical pathways have been shown, and cancer-associated lipid metabolism is to date an element of debate (8).

The Peng et al. investigated how to exploit altered fatty acid metabolism for prognostic purposes in colorectal cancer (CRC) patients. They correlated different gene expression profiles to different tumor stages and prognoses. Using differentially expressed lncRNAs, miRNAs and fatty acid metabolism-related mRNAs in CRC samples and adjacent normal samples they constructed for the first time a fatty acid metabolism-related competing endogenous RNA (ceRNA) network through databases predicting lncRNAs-miRNA and miRNA-mRNA interaction. The lncRNAs comprised in the newly ceRNA network were analyzed in order to assess their relation with OS and eight (AC156455.1, AC011462.4, TSPEAR-AS2, AL137782.1, LINC01857, ALMS1-IT1, AC022613.2, AC022144.1) were selected to build a prognostic signature for CRC which was validated in an internal and external cohort. Among the 8 lncRNAs, TSPEAR-AS2 was chosen for functional studies that demonstrated its involvement in fatty acid regulation.

The scientific challenge to define the role of reprogramming of energy metabolism in GC is an urgent need due to the poor prognosis of this neoplasm, despite the development of new strategies of treatment in recent years (3). Understanding such a phenomenon might allow to increase patients’ survival and to better understand the complex mechanisms that could explain the failure of several chemotherapeutic or biological agents (9). Bin et al. examined several modalities of metabolic reprogramming in GC, disclosed the intimate connection between cancer cells and tumor microenvironment (TME), the role of metabolic biomarkers as predictive factors of response, and the importance of tumor immunity cells. Bin’s team described the networks involved, and the drug-resistance mechanisms, focusing on the possibility to target the metabolic reprogramming to overcome resistances, as a “Trojan Horse” combinational strategy. They defined the role of immune cells and the preclinical evidence that is possible to improve the immune checkpoint inhibitors’ (ICIs) efficacy by combining them with compounds aimed to interfere with the metabolic and immune cycle of cancer cells. This is a crucial topic for oncological community: in fact, in the next future, the treatment of GC could be changed with immunotherapy, and if metabolic treatments will demonstrate to improve ICIs’ efficacy, especially by modulation of immune TME, the “metabolic” prospective could be the key to develop new potential treatment strategies.

As chronic hepatitis B and C viruses (HBV/HCV) are commonly found even in extra-hepatic tissues, Yang et al. aimed to demonstrate if the positivity to such etiologic agents might be correlated with gastric cancer (GC) pathogenesis. Indeed, they were able to observe a close positive association by analyzing four large databases (MEDLINE, EMBASE, Cochrane Library, and the PsychINFO) in a complex systematic review. Even though the biological mechanism on the direct involvement of HBV/HCV in GC pathogenesis is still elusive, Yang’s group contribution may pave the way to new important studies aimed to investigate the consequence of the viral infection in the gastric mucosa. Chronic inflammation as well as systemic impairment of immune function, liver cirrhosis, and the direct effects of HBV/HCV proteins on oncogenesis and/or on tumor suppression genes might play a major role in gastric carcinogenesis.

Moreover, the redox biology network applied to the pathophysiology and therapeutics of solid tumors demonstrated a sophisticated network of intrinsic and extrinsic cues, integrated into the tumor niche, driving tumorigenesis and tumor progression. Critical mutations and distorted redox signaling pathways orchestrate pathologic events inside cancer cells, resulting in resistance to stress and death signals, aberrant proliferation, and efficient repair mechanisms. Dang et al. developed for this purpose a risk model for prognosis in CRC patients *via* the identification of gene signatures driven by redox-related signaling pathways (redox-driven prognostic signature - RDPS). Indeed, they screened various pathways by analyzing the differences between tumors and controls in TCGA-CRC, determining that the vast majority of redox-related pathways were deeply dysregulated in CRC. Further, they established and validated a two-gene signature including ADH5 and HADH which are members of the alcohol dehydrogenase family, metabolizing a wide variety of substrates and likely to have a latent role in the malignant biological behavior of CRC. The RDPS resulted to be an independent risk factor for patient survival in four public cohorts and one clinical in-house cohort, demonstrating to be a potential clinical prospect in the prediction of metastasis and prognosis in CRC patients, contributing to the implementation of clinical decision-making. They also investigate if it might be helpful for the study of clinical immunotherapy regimens and find that in high-risk patients the risk score was inversely associated with local immunogenicity. In conclusion, the study of Dang et al. may shed new light on prognosis prediction and provide more promising evidence for immunotherapy in CRC.

## Author Contributions

All authors listed have made a substantial, direct and intellectual contribution to the work, and approved it for publication.

## Conflict of Interest

The authors declare that the research was conducted in the absence of any commercial or financial relationships that could be construed as a potential conflict of interest.

## Publisher’s Note

All claims expressed in this article are solely those of the authors and do not necessarily represent those of their affiliated organizations, or those of the publisher, the editors and the reviewers. Any product that may be evaluated in this article, or claim that may be made by its manufacturer, is not guaranteed or endorsed by the publisher.
